# Influence of Women´s Residence Region on Assisted Reproduction Treatments - Experience of a Tertiary Center in Northern Portugal

**DOI:** 10.5935/1518-0557.20210059

**Published:** 2022

**Authors:** Diana Rodrigues-Martins, Emídio Vale-Fernandes, Carla Leal, Márcia Barreiro

**Affiliations:** 1 Centro de Procriação Medicamente Assistida/Banco Público de Gâmetas, Serviço de Ginecologia, Departamento da Mulher e da Medicina Reprodutiva, Centro Materno-Infantil do Norte (CMIN), Centro Hospitalar Universitário do Porto (CHUP), Porto, Portugal; 2 Unidade Multidisciplinar de Investigação Biomédica (UMIB), Instituto de Ciências Biomédicas Abel Salazar da Universidade do Porto (ICBAS-UP), Porto, Portugal

**Keywords:** infertility, social determinants of health, *in vitro* fertilization, demography, residence characteristics

## Abstract

**Objective:**

Data on the possible influence of women´s region of residence, within the same country, on the outcomes of medically assisted reproduction cycles are scarce. This study aims to assess the impact of the women's region of residence on the results of *in-vitro* fertilization cycles.

**Methods:**

We evaluated *in-vitro* fertilization cycles between 2010 and 2017, performed in a northern Portugal assisted reproduction center. We defined two groups: Douro Litoral (group 1; n=783), and Trás-os-Montes and Alto Douro (group 2; n=178). We analyzed demographics and cycle-related variables, and we calculated the rates for embryo transfer cycles. We used the Mann-Whitney and Chi-square tests and *p*<0.05 was considered statistically significant.

**Results:**

We included 961 cycles. The region of residence had no effect on the following variables: women´s age; body mass index; or duration of infertility (*p*>0.05). Group 2 had a statistical significant lower number of previous cycles than group 1 (1.3±0.5 and 1.5±0.7; *p*=0.005). In the sub-analysis of cycles with embryo transfer (n=781), group 1 obtained had rates of normal fertilization (62.5% *vs*. 57.5%; *p*=0.04), miscarriage rate (30.0 *vs*. 10.9%; *p*=0.007), and lower implantation rates compared to group 2 (33.3% *vs*. 50.0%; *p*<0.001).

**Conclusions:**

Women from the region of Trás-os-Montes e Alto Douro had a lower number of previous cycles, compared to those from the Douro Litoral, despite the absence of statistical significant differences in terms of age or infertility duration. These findings reinforce the need to contemplate the sociodemographic and socioeconomic variables in this context.

## INTRODUCTION

Infertility is defined by the National Survey of Family Growth (NSFG) as the failure to conceive after at least 12 consecutive months of unprotected sexual intercourse (Direcção-Geral da Saúde, 2011). This condition remains a major public health concern worldwide, given its social and economic burden (Direcção-Geral da Saúde, 2011). The biggest study on this subject to date in Portugal estimates a prevalence of 9% (Carvalho & Santos, 2009).

A systematic review published in 2017 on worldwide trends in assisted reproductive technology (ART) between 2004-2013 demonstrated considerable disparities in live birth rates between seven different regions in the world (Kushnir *et al*., 2017). The ART results depend on diverse factors, not only epidemiological (the woman's age, the causes of infertility, etc.) and the clinical practice adopted (pharmacological treatments, the policy regarding embryo transfer, etc.), but also on the social context in which such techniques are applied (Hearns-Stokes *et al*., 2000). Indeed, social determinants of health such as low income, disparities in access to care, and minority status are considered stressors, and have been related to poor outcomes of care, both within and outside of the context of infertility (Hansen *et al*., 2016).

The region where the patient lives has an influence on lifestyle behaviors (Domar *et al*., 2015). However it is unclear whether the region where the patient lives within the same country, and consequently under the same public insurance law, would influence ART usage and its outcomes. This study aims to assess if the region where the women live would predict ART usage and treatment outcomes. Our goal is to assess whether or not knowledge about this subject may enable more effective counseling and treatment planning, as well as guide national policies on social determinants of health.

## MATERIALS AND METHODS

**Figure 1:**
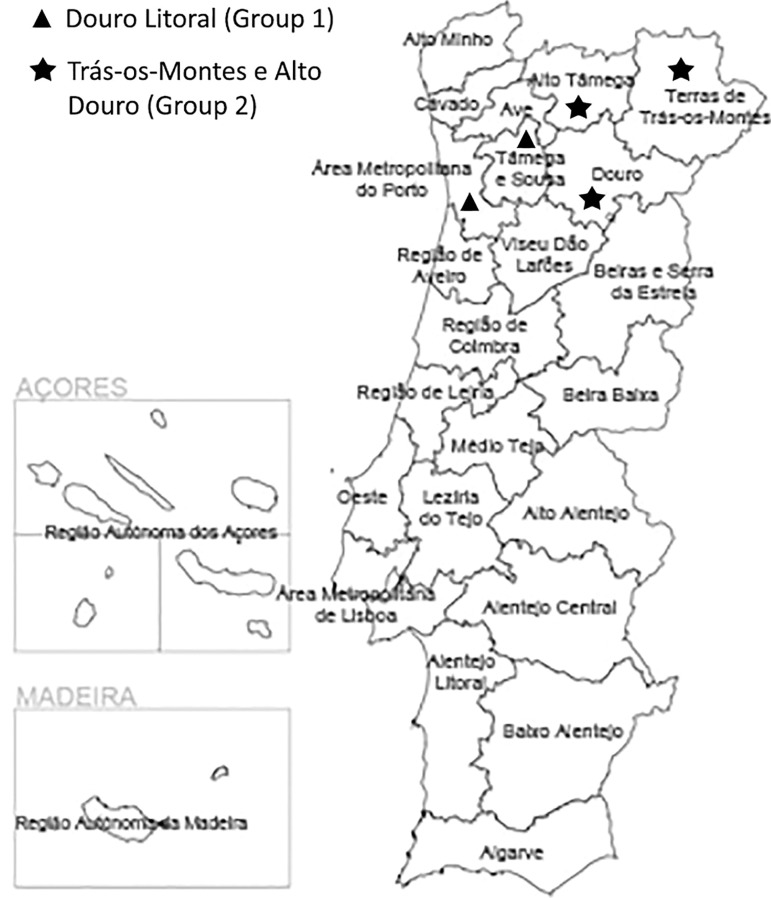
Reference areas.

We designed a retrospective study to examine the impact of the woman's region of residence on the results of *in-vitro* fertilization (IVF) cycles between 2010 and 2017, from a northern Portugal tertiary public center - Centro Materno Infantil do Norte, Centro Hospitalar Universitário do Porto. The reference area of this center encompasses two main regions: Douro Litoral, constituted by the Metropolitan area of Porto e Tâmega e Sousa, and Trás-os-Montes e Alto Douro, constituted by the Douro, Alto Tâmega and Terras de Trás-os-Montes. Figure 1 depicts a visual representation of this reference map. Supplementary Table 1 shows the official national data on socioeconomic characterization of these regions.

The center's practice during the study period followed the recommendations from the national healthcare system for public centers, which grants free access to the IVF procedure itself up to three attempts, as well as the medical appointments, and a substantial co-payment on the costs of drugs used for ovarian stimulation. Having more than forty years at follicular puncture is the maternal ceiling (Diário da República, 2006; 2009). The authors received institutional approval for the study.

After excluding the cycles using donor oocytes, 961 IVF cycles (including the "classical" IVF as well as intracytoplasmic sperm injection procedure) were performed during the study period. We defined two groups for comparison: Douro Litoral (Group 1; n=783), and Trás-os-Montes and Alto Douro (Group 2; n=178). We took demographic and cycle-related variable information from the patients' files.

We then analyzed several outcomes for cycles, which did not end up being cancelled. The outcomes for this study included: fertilization rate (fertilized oocytes/inseminated or injected oocytes); pregnancy rate (clinical pregnancies/transferred patients); implantation rate (embryos in ultrasound/embryos transferred); miscarriage rate (abortions before 20 gestational weeks/ clinical pregnancies).

We used the SPSS^®^ version 21 for statistical analyzes- the Mann Whitney and Chi-square tests. A value of *p*<0.05 was considered statistically significant.

## RESULTS

We had 961 IVF cycles meeting the inclusion criteria. Group 1 consisted had 783 cycles performed on women living in Douro Litoral, and group 2 consisted of 178 cycles performed on women living in Trás-os-Montes e Alto Douro (Table 1).

**Table 1.** Patient and ovarian stimulation characteristics by women´s region of residence.

Variable	Region (n=961 cycles)		*p*-value^a^
	**Douro Litoral (n=783)**	**Trás-os-Montes e Alto Douro (n=178)**	
Age (years)	35±3.7 (20-40)	34±3.6 (24-40)	0.493
Body Mass Index (Kg/m2)	23.8±4.2 (16.2-41.7)	24.2±4.7 (16.0-41.1)	0.345
Duration of infertility (years)	4±2.1 (1-16)	4.5±2.9 (1-16)	0.170
Number of previous cycles	1.5±0.7 (1-4)	1.3±0.5 (1-3)	**0.005**
Total dose of gonadotropin (IU)	2318±1498 (200-18000)	1938±898 (600-5400)	**0.003**
Duration of ovarian stimulation (days)	9.1±1.9 (4-17)	8.9±1.7 (6-15)	0.097
Cycles without embryo transfer	140 (17.9%)	40 (22.5%)	0.156

Values are mean±standard deviation (minimum-maximum), or number (percentage) unless otherwise stated.

a Mann–Whitney U test or Chi-square test. A *p*<0.05 was considered statistically significant.

Analyzing patient demographics, there were no differences on the following variables: women's age, body mass index, or duration of infertility (*p*<0.05), according to the region of residence (Table 1). When it came to infertility duration and number of previous IVF cycles, women in group 2 had been infertile for a longer time (4.5±2.9 years *versus* 4.0±2.1 years for women in group 2 and 1, respectively; *p*=0.170), and had performed significantly fewer IVF attempts (1.3±0.5 in group 2 compared to 1.5±0.7 in group 1; *p*=0.005) (Table 1). When it came to ovarian stimulation characteristics, the total dose of gonadotropin and duration of ovarian stimulation were higher for group 1 (Table 1; *p*=0.003 and *p*=0.097). No embryo was transferred in about one-fifth of the women in each group, more precisely 140 women (17.9%) in group 1 and 40 (22.5%) in group 2; *p*=0.156 (Table 1).

Group 1 had 643 (82.3%) of the 781 cycles in which embryo transfer was performed. Table 2 shows cycles outcomes. Fertilization rates were significantly higher in group 1 compared to group 2 (62.5% and 57.5%; *p*=0.04). The pregnancy rate had the same trend; however, the difference between group 1 and 2 did not reach statistical significance (35.5% *vs.* 34.1%; *p*=0.745). On the other hand the implantation rate was significantly higher for cycles performed by group 2 (33.3% and 50.0% for group 1 and 2, respectively; *p*<0.001). Miscarriage was more prevalent among women in group 1 compared to those in group 2 (30.0% *vs.* 10.9%; *p*=0.007).

**Table 2.** Characteristics of the cycles with embryo transfer by women´s region of residence.

Variable	Total (n=781 cycles)	Region		*p*-value^a^
		**Douro Litoral**	**Trás-os-Montes e Alto Douro**	
Number of cycles	781 (100%)	643 (82.3%)	138 (17.7%)	NA
Fertilization rates (%)	61.5	62.5	57.5	0.04
Pregnancy rates (%)	35.7	35.5	34.1	0.745
Implantation rates (%)	33.3	33.3	50.0	< 0.001
Abortion rates (%)	26.3	30.0	10.9	0.007

Values are number (percentage) or percentage unless otherwise stated. NA - Non-applicable.

a Mann–Whitney U test or Chi-square test. A *p*<0.05 was considered statistically significant.

## DISCUSSION

Whereas much has been written about the prognostic factors associated with IVF outcomes, such as female age, diagnosis, and ovarian reserve, relatively little attention has been dedicated to patient-oriented lifestyles and social determinants of health, that may influence IVF outcomes as well (Hornstein, 2016; Berga, 2016). The majority of the sparse literature published on this subject make comparisons between countries, where differences in healthcare policies influence the results (European IVF-monitoring Consortium, 2017; Präg & Mills, 2017).

In this study, there were disparities when it came to treatment usage and its outcomes depending on women´s region of residence, within the same country and at the same ART center. Since access to fertility treatments in Portuguese national health system is comprehensive, insurance coverage for fertility care did not explain the differences observed. Women from the region of Trás-os-Montes e Alto Douro (group 2) had a lower number of previous treatment cycles, compared to those from the Douro Litoral (group 1), despite the absence of significant differences in terms of age or infertility´s duration. It is still worth mentioning that, although it did not reach statistical significance, women in the group 2 had longer duration of infertility, compared to their counterparts in group 1. When analyzing the national data on social economic status (SES), Tras-os-Montes e Alto Douro is reported to have less local access to college education, and lower income compared to Douro Litoral (Pordata, 2020). These findings are in agreement with previously published data from Kessler *et al*., who found that education was positively associated with seeking fertility evaluation; and income was positively associated with treatment (Kessler *et al*., 2013). In another study, lower education was a predictor of lower treatment seeking, even in a country with publicly funded access to fertility treatments (Schmidt, 2006). A correlation between a woman's educational level and understanding the cycle instructions has already been suggested (Mahalingaiah *et al*., 2011). Since we observed greater odds of cycle cancellation among women in group 2, we can speculate that education itself may cause successful progression to egg retrieval. The age factor must also be taken into account on this outcome.

If it seems obvious that educational level is linked to a higher possibility of recognizing fertility problems and seeking help (Costa *et al*., 2013), it´s not that plain to explain the differences in treatments usage when health insurance is not a problem. Dieke *et al*. (2017) support that while a comprehensive insurance coverage may increase access and address some or all of the cost barriers, strategies that address other cost and non-cost factors may be needed to help eliminate utilization disparities. The Portuguese law grants access to IVF procedure itself free of charge and a high contribution to paying for the drugs; however, factors such as travel expenses are not addressed (Diário da República, 2006; 2009). This may be an issue for couples in Trás-os-Montes e Alto Douro, since the majority of the tertiary centers performing IVF are located in the Douro Litoral.

Having a higher education has been associated with a better overall health status independent of age, and consequently higher pregnancy rates (Smith *et al*., 2011). There is growing evidence that lifestyle habits can have a significant impact on the outcome of the advanced reproductive technologies, and women's habits vary within the same country (Domar et al., 2015). Despite having a higher fertilization and pregnancy rate, Douro Litoral also presented with a higher miscarriage rate. In the future we think it would be interesting to explore possible explanations for these differences, approaching not only women´s health habits, such as smoking and physical exercise, but environmental exposure as well, since Douro Litoral is a much more industrialized region compared to Trás-os-Montes e Alto Douro.

To our knowledge, this is the first publication to evaluate the impact of women´s region of residence within an European country in the context of fertility care. The large sample size coming from only one center, where the same practices applies is a strength to this study. However, this study is not without limitations. First, our results relate exclusively to cases who did not find a solution before being referred to a tertiary center. Thus, the design of the study does not provide the opportunity to understand whether the demonstrated differences are related to potential barriers to access to tertiary level infertility centers. Second, it would be important to take into account several variables, such as education degree, income, smoking habits or previous miscarriage, which are important for the statistical comparison to control for confounding factors. Third, pregnancy and live birth rates have been the traditional metrics of ART success. Forth, couples referred to tertiary centers usually have longer duration of infertility and are more likely affected with more than one, or more severe fertility factors than those with a shorter infertility duration, so generalizations must be made with caution. Finally it should be mentioned that culture and religion, traditionally more conservative in the east side of the country, may have an important influence on this subject.

## CONCLUSIONS

In conclusion, our data suggests that the availability of affordable fertility treatments is necessary but not sufficient, and adds to the perspective that SES is an important driver in seeking evaluation and treatment. This acknowledgement should prompt us to look beyond the obvious question about which agent is best for ovarian stimulation, to the increasingly important topic of social determinants of health.

## Appendix

**Supplementary Table 1.** Socioeconomic data for regions of interest in 2018 according to the national statistics.

	Douro Litoral (group 1)		Trás-os-Montes e Alto Douro (group 2)		
	**Metropolitan area**	**Tâmega e** **Sousa**	**Alto** **Tâmega**	**Douro**	**Terras de Trás-os-Montes**
Resident population	1.721.038	418018	86812	191574	108204
Territory area (Km^2^)	2.041.3	18315	2921.9	4031.6	5543.6
Average monthly earnings of employees (euros)	1165	858	941	636	918
Unemployed persons registered in employment centers as a % of the resident population with 15 to 64 years	7%	6%	7%	9%	6%
Number of colleges	62	2	1	6	5
Number of students in college *	75.930	1595	189	6.896	7.229

* The year presented corresponds to the last year of the academic year pair. Available in https://www.pordata.pt
